# Acute Cardioembolic and Thrombotic Middle Cerebral Artery Occlusions Have Different Morphological Susceptibility Signs on T2^*∗*^-Weighted Magnetic Resonance Images

**DOI:** 10.1155/2015/839820

**Published:** 2015-10-12

**Authors:** Mei Zheng, Dong-sheng Fan

**Affiliations:** Department of Neurology, Peking University Third Hospital, Beijing 100191, China

## Abstract

Presence of susceptibility sign on middle cerebral artery (MCA) in T2^*∗*^-weighted magnetic resonance (MR) images has been reported to detect acute MCA thromboembolic occlusion. However, the pathophysiologic course of thrombotic MCA occlusion differs from embolic occlusion, which might induce different imaging characters. Our study found that the occurrence rate of the MCA susceptibility sign in cardioembolism (CE) patients was significantly higher than in large artery atherosclerosis (LAA) patients, and the diameter of the MCA susceptibility sign for CE was greater than for LAA. Moreover, the patients with hemorrhagic transformation had MCA susceptibility signs with a significant larger mean diameter than patients without hemorrhagic transformation. Therefore, we hypothesized that the morphology of susceptibility signs could be used to differentiate acute cardioembolic and thrombotic MCA occlusions, which helped to select appropriate treatment strategies for different patients.

## 1. Introduction

T2 Previous studies have reported that the middle cerebral artery (MCA) susceptibility sign in T2^*∗*^-weighted magnetic resonance (MR) images can be indicative of an acute thromboembolic occlusion [1–5]. Acute thrombi and emboli contain large amounts of deoxygenated hemoglobin, which can severely shorten T2-weighted signals. This magnetic susceptibility effect produces a nonuniform magnetic field and a rapid dephasing of proton spins, which result in signal loss that is best observed on T2^*∗*^ susceptibility-weighted images [6]. The MCA susceptibility sign presents as a significantly decreased signal within an MCA trunk that exceeds the normal vessel diameter [6]. The hyperdense MCA sign (HMCAS) is a well-established marker of early ischemia on noncontrast computed tomography (CT). Recent studies show that the MCA susceptibility sign is more sensitive in predicting acute thromboembolism than HMCAS in CT [7] and can be used to predict the immediate effectiveness of intra-arterial thrombolysis [3].

Cardioembolic (CE) stroke is usually associated with high mortality as it has larger infarct size and higher rate of hemorrhage transformation compared to thrombotic occlusion. The etiology of ischemic stroke affects prognosis, outcome, and management. Traditionally, we distinguish CE from large artery atherosclerosis (LAA) through information provided by the speed of onset, history of atrial fibrillation, and infarct size in imaging data. However, whether there are direct signs to differentiate CE from LAA has not been well studied. The objective of this study is to explore the differences in MCA susceptibility sign between emboli and thrombi.

## 2. Methods

### 2.1. Subjects

**Figure 1:**
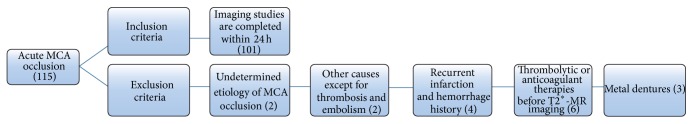
Patients selection flowchart.

From August 2010 to November 2012, 115 patients with acute MCA occlusion were admitted to our institution. The inclusion criteria for acute MCA occlusion patients were as follows: (1) all of the patients met the 2010 Chinese guidelines for the diagnosis and management of acute ischemic stroke [8]; (2) acute MCA occlusion was the first attack; (3) the diffusion-weighted image (DWI), T2^*∗*^-weighted MR image, and head and neck MR angiogram (MRA) were completed within 24 hours of symptom onset; and (4) DWI confirmed that the infarct was distributed in the unilateral MCA area. The exclusion criteria were as follows: (1) there are recurrent cerebral infarction and past cerebral hemorrhage; (2) MCA occlusion is caused by Moyamoya disease, arterial dissection, vasculitis, tumor, and blood hypercoagulability, among other causes; (3) thrombolytic or anticoagulant therapies were performed prior to the imaging examination; (4) MRA confirmed that the internal carotid artery contained moderate stenosis or occlusion; and (5) metal dentures affected T2^*∗*^-weighted MR image quality. Eight-four patients met these criteria eventually (Figure 1). Among these 84 patients, there were 63 men (age range, 41–89 years; mean age, 63.6 years) and 21 women (age range, 47–86 years; mean age, 64.4 years). Our institutional review board approved this study, but patient informed consent was not required because this study was retrospective.

The patients were divided into two groups according to the Trial of Org 10172 Acute Stroke Treatment (TOAST) criteria [9]: CE and LAA. CE patients had classic clinical manifestations, including a previous TIA or stroke in more than one vascular territory or systemic embolism, together with high-risk and medium-risk cardiac source for the embolus. LAA patients had history of intermittent claudication, TIA in the same vascular territory, and evidence of large artery arthrosclerosis other than responsible vessel. There were 18 (21%) patients in CE and 66 (79%) patients in LAA.

Stroke severity was assessed by using the National Institutes of Health Stroke Scale (NIHSS). All stroke severity examinations were performed at admission and 1 week after onset by a stroke neurologist. The mean NIHSS score at admission was 11.3 (range, 4–25) and 1 week later 9.1 (range, 0–25).

### 2.2. MRI/MRA Protocol

All patients were examined on a 1.5-T MRI unit (Sonata, SIEMENS, Erlangen, Germany). For T2^*∗*^-weighted MRI, the slice thickness was 5 mm, and the intersection gap was 2 mm. For DWI using an echo-planar sequence, the slice thickness was 5 mm, and the *b* values were 0 and 2,000 s/mm^2^. Additional parameters for the acute stroke MR protocol were as follows: axial fast spin-echo T2-weighted MRI (repetition time (TR), 3700 ms; echo time (TE), 95 ms; excitations, 1; slice thickness, 5 mm; slice gap, 1 mm; matrix size, 256 × 256; field of view, 240 mm) and 3-dimensional time-of-flight MR angiographic images (TR, 39 ms; TE, 6.5 ms; excitations, 1; slab thickness, 60 mm; partitions, 60; matrix size, 160 × 512; field of view, 200 mm).

### 2.3. Image Analysis

**Figure 2:**
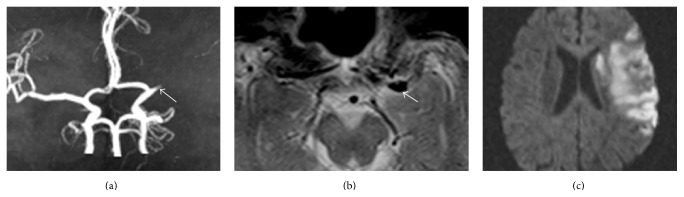
The MCA susceptibility sign in CE patients. (a) MRA reveals an MCA occlusion. (b) T2^*∗*^-weighted MR imaging indicates a large diameter and a short, low-signal image along the MCA. (c) DWI shows a large-acreage cerebral infarction.

**Figure 3:**
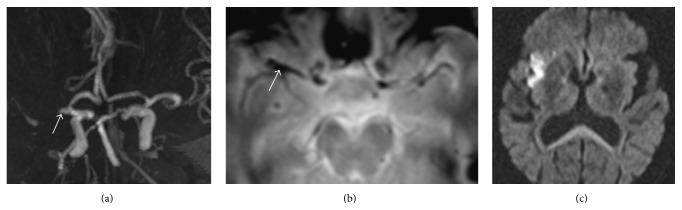
The MCA susceptibility sign in LAA patients. (a) MRA reveals an MCA occlusion. (b) T2^*∗*^-weighted MR imaging indicates a low-signal image with a relatively small diameter and long length along the MCA. (c) DWI shows that the infarct size was relatively small compared to CE patients.

The imaging data of all selected patients met consensus reading by two experienced neuroradiologists Sun A-P. and Sun Q.-L. who were blinded to the patients' clinical information. MRI criteria for MCA susceptibility sign were defined as low-signal images along the blood vessel and exceeding vessel diameter of the MCA [7], especially compared to the contralateral artery (Figures 2 and 3). The signs were judged to be present on the basis of only unanimous decision. If a disagreement had occurred, the sign would have been judged as absent. The diameter, length, occurrence, and location (M1 or M2) of the low-signal images were analyzed. The measurement method used ImageJ software (Version 1.46, NIH, Bethesda, MD, USA, http://rsb.info.nih.gov/ij/index.html). Infarct size was expressed as a percentage of the high-signal area in DWI and the area of ipsilateral hemisphere.

### 2.4. Statistical Analysis

The Statistical Package for Social Sciences (SPSS version 16.0, SPSS Inc., USA) was used for statistical analysis. Numeric values were expressed as the means ± standard deviations (SD), and categorical variables were expressed as percentages. The independent-samples *t*-test was used to compare the numeric values between CE and LAA. The Pearson Chi-Square test was used to evaluate the categorical data between the two groups. A value of *P* < 0.05 was considered statistically significant.

## 3. Results

### 3.1. Clinical Characteristics of CE and LAA Patients with Acute MCA Occlusions

Among the 84 patients studied, there were 18 patients in CE and 66 in LAA. The differences between the two patient groups in terms of patient sex (male/female, 14/4 versus 69/17), history of hypertension (61.1 versus 68.2%), diabetes (33.3% versus 28.8), hyperlipidemia (66.7% versus 54.6%), or smoking (27.8% versus 36.4%) were not statistically significant (*P* > 0.05). On average, CE patients were significantly older than LAA patients (70.5 ± 5.9 years versus 62.3 ± 11.2 years, *P* < 0.01) and had a higher proportion of history of coronary heart disease (50.0% versus 22.7%, *P* < 0.05). CE patients had a significantly higher NIHSS score than LAA patients at admission (14.8 ± 5.3 versus 10.3 ± 4.6, *P* < 0.01) and after 1 week (12.2 ± 6.3 versus 8.3 ± 5.7, *P* < 0.05) (Table 1).

### 3.2. Different Morphologies of MCA Susceptibility Signs in CE and LAA Patients

Among the 84 acute MCA occlusion patients, 63 (75%) presented an MCA susceptibility sign. A greater number of CE patients were positive for the MCA susceptibility sign compared to LAA patients (100% versus 68.2%, *P* < 0.01). Compared with LAA patients, CE patients had larger-diameter low-signal images at the occluded artery (5.4 ± 0.9 mm versus 3.9 ± 1.0 mm, *P* < 0.01), but the lengths were shorter (11.2 ± 2.2 mm versus 16.6 ± 4.2 mm, *P* < 0.01) (Figures 2(b) and 3(b)). The cut-off value were 4.385 mm and 14.89 mm, respectively. M1 was the most common site of positive MCA susceptibility sign without difference between two groups (M1/M2, 14/4 versus 36/9, *P* > 0.05). Compared with LAA patients, CE patients had significantly larger infarct areas (32.6 ± 21.5% versus 18.4 ± 12.7%, *P* < 0.01) and a higher rate of hemorrhagic transformation (27.8% versus 7.6%, *P* < 0.05) (Table 2).

In 63 cases with a positive MCA susceptibility sign, the diameters of the low-signal images in patients with hemorrhagic transformation were significantly larger than in those without hemorrhagic transformation (5.4 ± 0.9 mm versus 4.1 ± 1.2 mm, *P* < 0.01), although their lengths did not differ (14.4 ± 3.4 mm versus 15.2 ± 4.7 mm, *P* > 0.05).

## 4. Discussion

In this study, we find that emboli and thrombi in acute occlude MCA have different shape of susceptibility sign in T2^*∗*^-MRI. The susceptibility sign of emboli has a larger diameter and relatively shorter length than thrombi, and large diameter of susceptibility sign is related to hemorrhage transformation.

In this study, T2^*∗*^-weighted MRI can sensitively detect acute thromboembolic MCA occlusion. 75% patients among 84 acute MCA occlusions have a positive susceptibility sign. HMCAS on CT was thought to be the important early changes in acute ischemic stroke of MCA territory previously [10, 11]. However, a recent study indicates that 3D susceptibility-based perfusion MRI allows the identification of acute MCA thromboembolism with a sensitivity higher than that of CT [7]. Compare with LAA, CE patients have a higher occurrence rate of positive MCA susceptibility signs in our study (18 of 18, 100%, versus 45 of 66, 68.2%). Kim et al. [3] drew the same conclusion, as 13 of 16 patients with a positive MCA susceptibility sign had a history of atrial fibrillation and no evidence of LAA stenosis by digital subtraction angiography. Cho et al. [12] also concluded that MCA susceptibility is an independent predictor of CE stroke. In their data, MCA susceptibility was more commonly associated with CE stroke patients (31 of 40, 77.5%) than with other stroke subtypes (14 of 55, 25.5%). However, the rate of susceptibility sign in LAA might be underestimated in these studies, as MCA stenosis sometimes were exaggerated as occlusion in MRA which would induce false-negative results of MCA susceptibility sign more easily in LAA patients.

Different composition of thromboemboli in CE and LAA may create different shape of susceptibility sign although deoxygenated hemoglobin in the thromboemboli is the main material producing susceptibility sign. Cardiogenic emboli are primarily composed of red thrombi, which contain numerous erythrocytes and some fibrin. Intra-arterial primary clots, however, are mainly composed of white thrombi that consist of varying amounts of cellular debris, fibrin, and platelets, but only a small number of red blood cells [13–15].

An important finding of this study is that the MCA susceptibility sign in CE patients is larger in diameter than that found in LAA patients. There is strong evidence in our study that the large diameters of MCA susceptibility signs are associated with hemorrhagic transformation. The destruction of the extracellular matrix integrity is considered to be the cause of hemorrhagic transformation, especially parenchymal hemorrhage after acute infarction [16, 17]. However, arterial wall destruction is hard to observe by current imaging technology. A recent study reported that increased permeability is present in the basal ganglia region in patients with parenchymal hemorrhage after acute stroke [18]. As the bleeding site is close to the MCA trunk, the destruction of the focal arterial wall might be the anatomic basis of the parenchymal hemorrhage.

For acute thromboembolic MCA occlusions, the optimal treatment option is thrombolysis [19]. However, the symptoms deteriorate once hemorrhagic transformation occurs after intravenous thrombolysis. The selection of appropriate candidate patients for thrombolysis is vitally important for a favorable prognosis. Atrial fibrillation has been testified as the independent risk factors for symptomatic intracranial hemorrhage and no early recanalization after thrombolysis [20, 21]. Our study indicates that larger diameters of MCA susceptibility sign often present in CE patients and accompany more hemorrhage transformation. Thus, the shape of thromboemboli in T2^*∗*^-MR imaging can help us to manage patients with acute MCA occlusion. Recent study reported that HMCAS length >10 mm or persistence of HMCAS on follow-up CT scan was associated with poor outcome after intravenous thrombolysis and needed ancillary therapy [22, 23]. Susceptibility vessel sign has been used to predict artery recanalization [24] and metabolic state of infarcted brain tissue [25].

The limitation of our study is that the cases did not have a unified treatment plan. Thus we cannot come to a conclusion about the effect of MCA susceptibility to the prognosis of stroke treatment. Our next work will aim at the predictive role of the shape of MCA susceptibility sign on outcomes of intravenous thrombolysis in CE and LAA patients.

## 5. Conclusion

The morphology of susceptibility signs on T2^*∗*^-weighted MR images can be used to differentiate acute cardioembolic and thrombotic MCA occlusions. The larger diameters of MCA susceptibility signs in CE patients indicate a greater probability of hemorrhagic transformation after acute cerebral infarction.

## Figures and Tables

**Table 1:** Clinical manifestations and risk factors of CE and LAA patients with acute MCA occlusions.

Types	Total (*n* = 84)	CE (*n* = 18)	LAA (*n* = 66)	*t*/*χ* ^2^ values	*P* values
Sex (male/female)	63/21	14/4	49/17	*χ* ^2^ = 0.094	0.762
Mean age (years, mean ± SD)	64.1 ± 10.8	70.5 ± 5.9	62.3 ± 11.2	*t* = 2.975	0.004
Hypertension history (*n*, %)	56 (66.7)	11 (61.1)	45 (68.2)	*χ* ^2^ = 0.318	0.578
Diabetes history (*n*, %)	25 (29.8)	6 (33.3)	19 (28.8)	*χ* ^2^ = 0.140	0.713
Coronary heart disease history (*n*, %)	24 (28.6)	9 (50.0)	15 (22.7)	*χ* ^2^ = 5.155	0.023
Hyperlipidemia history (*n*, %)	48 (57.1)	12 (66.7)	36 (54.6)	*χ* ^2^ = 0.848	0.363
Smoking history (*n*, %)	29 (34.5)	5 (27.8)	24 (36.4)	*χ* ^2^ = 0.461	0.503
NIHSS score at admission (mean ± SD)	11.3 ± 5.0	14.8 ± 5.3	10.3 ± 4.6	*t* = 3.585	0.001
NIHSS score at 1 week later (mean ± SD)	9.1 ± 6.0	12.2 ± 6.3	8.3 ± 5.7	*t* = 2.513	0.014

**Table 2:** The characteristics of MCA susceptibility signs in CE and LAA patients.

Types	Total (*n* = 84)	CE (*n* = 18)	LAA (*n* = 66)	*t*/*χ* ^2^ values	*P* values
MSS positives^**∗**^ (%)	63 (75.0)	18 (100)	45 (68.2)	*χ* ^2^ = 7.636	0.005
Diameter of MSS (mm)	4.1 ± 1.0	5.4 ± 0.9	3.9 ± 1.0	*t* = 5.567	0.000
Length of MSS (%)	15.1 ± 4.5	11.2 ± 2.2	16.6 ± 4.2	*t* = −5.135	0.000
Location of MSS (M1/M2)	50/13	14/4	36/9	*χ* ^2^ = 0.039	0.847
Infarct area (%)	21.5 ± 16.0	32.6 ± 21.5	18.4 ± 12.7	*t* = 3.577	0.001
Hemorrhage transformation (%)	10 (11.9)	5 (27.8)	5 (7.6)	*χ* ^2^ = 5.504	0.019

^*∗*^MSS: MCA susceptibility sign.
